# A self-controlled case series study to measure the risk of SARS-CoV-2 infection associated with attendance at sporting and cultural events: the UK Events Research Programme events

**DOI:** 10.1186/s12916-024-03276-4

**Published:** 2024-03-06

**Authors:** Ian J. Douglas, Jerlyn Peh, Kathryn E. Mansfield, Anna Trelfa, Tom Fowler, Matthew Boulter, Paul Cleary, Jenifer Smith, W. John Edmunds

**Affiliations:** 1 London School of Hygiene and Tropical Medicine, London, UK; 2 UK Health Security Agency, London, UK; 3 William Harvey Institute, Queen Mary University of London, London, UK; 4 Atlantic Medical Group, Penzance, UK

**Keywords:** COVID-19 transmission, Mass events, Epidemiology

## Abstract

**Background:**

In 2021, whilst societies were emerging from major social restrictions during the SARS-CoV-2 pandemic, the UK government instigated an Events Research Programme to examine the risk of COVID-19 transmission from attendance at cultural events and explore ways to enable people to attend a range of events whilst minimising risk of transmission. We aimed to measure any impact on risk of COVID-19 transmission from attendance at events held at or close to commercially viable capacity using routinely collected data.

**Methods:**

Data were obtained on attendees at Phase 3 Events Research Programme events, for which some infection risk mitigation measures were in place (i.e. evidence of vaccination or a negative lateral flow test). Attendance data were linked with COVID-19 test result data from the UK Test and Trace system. Using a self-controlled case series design, we measured the within person incidence rate ratio for testing positive for COVID-19, comparing the rate in days 3 to 9 following event attendance (high risk period) with days 1 and 2 and 10–16 (baseline period). Rate ratios were adjusted for estimates of underlying regional COVID-19 prevalence to account for population level fluctuations in infection risk, and events were grouped into broadly similar types.

**Results:**

From attendance data available for 188,851 attendees, 3357 people tested positive for COVID-19 during the observation period. After accounting for total testing trends over the period, incidence rate ratios and 95% confidence intervals for positive tests were 1.16 (0.53–2.57) for indoor seated events, 1.12 (0.95–1.30) for mainly outdoor seated events, 0.65 (0.51–0.83) for mainly outdoor partially seated events, and 1.70 (1.52–1.89) for mainly outdoor unseated multi-day events.

**Conclusions:**

For the majority of event types studied in the third phase of the UK Events Research Programme, we found no evidence of an increased risk of COVID-19 transmission associated with event attendance. However, we found a 70% increased risk of infection associated with attendance at mainly outdoor unseated multi-day events. We have also demonstrated a novel use for self-controlled case series methodology in monitoring infection risk associated with event attendance.

**Supplementary Information:**

The online version contains supplementary material available at 10.1186/s12916-024-03276-4.

## Background

Infection with respiratory viruses is increased by social interactions. In 2021, as social restrictions due to the ongoing SARS-CoV-2 pandemic started to ease, it was unclear whether attendance at cultural and sporting events would be associated with an increased risk of SARS-CoV-2 transmission, above expected levels of community transmission. To investigate this further, the UK government’s Department for Digital, Culture, Media and Sport (DCMS) established an Events Research Programme (ERP) to examine the impact of attendance at a broad range of sport and cultural events on the risk of SARS-CoV-2 transmission and to explore ways of reducing transmission risk [1].

Between June and August 2021, the third phase of the ERP facilitated a wide range of sport and cultural events run at, or close to, full capacity with a specially established Science Board overseeing the programme. At the time, events at such capacity outside of the ERP were not legally permitted in England. The ERP commissioned a series of studies to examine the impact of events on SARS-CoV-2 transmission, attendee behaviour, and environmental/air quality at the events [2]. A condition of entry for all events was that attendees were asked to self-declare that they were symptom free and ensure that they had taken a lateral flow test (LFT) with a negative result within 48 h of arrival. In general, these LFTs were unsupervised and relied on a trust-based approach.

Here, we present the results of a study designed to measure whether event attendance was associated with an increased risk of infection with SARS-CoV-2 and whether this varied across different types of event [3]. The commissioning of this evaluation was led by the DCMS as part of the ERP. The Department of Health and Social Care Tests and Tracing infrastructure (now part of UK Health Security Agency; UKHSA) was used to support the programme.

## Methods

### Study population

Phase 3 ERP event organisers provided data, where available, on attendees, to the UKHSA. The study population was then formed from the subset of these attendees, who also had a result for any SARS-CoV-2 test recorded in the National Health Service (NHS) COVID-19 Test and Trace system in days 1–18 following event attendance. One of the remits of the Test and Trace system was to record SARS-CoV-2 test results in England, whether positive or negative. Data were available on attendee age, sex, the event attended, date of the event, and self-reported COVID-19 vaccine status. The proportion of attendees for whom attendance data were available varied between events for two reasons. Some events asked attendees to actively opt in to making data available, in which case-only data for those providing consent were made available. For other events, availability was determined by how organisers arranged and recorded bookings.

### Exposure, outcome, and covariates

The exposure was attendance at a phase 3 ERP event, as recorded by the event organisers. Events for which data were available are listed in Table 1. Events varied in nature substantially and have been broadly grouped according to predominant features: indoor seated, outdoor seated, outdoor partially structured, and outdoor unstructured. Categorisation was for convenience, and we recognise that, in reality, all events have a more complex nature. For multi-day events, where recorded, first and last possible attendance dates were obtained. Where exact attendance dates for multi-day events were unavailable (i.e. Latitude and Tramlines festivals), attendance on all dates was assumed.

**Table 1:** Breakdown of phase 3 events research programme events studied

Event type	Event	No. of days; date(s) of event	Estimated total ticketed attendees^a^ (single day events)/max attendees/event (multi-day events)	Attendee data available to UKHSA	Individuals available for study (data available on attendee and any + or − test result in test and trace within 18 days after event)	Rate ratio for negative test results
Indoor seated	Grange Festival^b^	10 days; 1–18 July 2021	596	1577	754	1.00 (0.90–1.10)
	A Little Night Music^b^	6 days (between 14 and 17 July 2021)	264	983	493	
	Shot of Laughter^c^	1 day; 17 July	650	218	152	
	Adam Kay^c^	1 day; 20 July	620	572	305	
	Rob Beckett^c^	1 day; 23 July	454	440	236	
Outdoor seated	Wimbledon^d^	14 days; 28 June–11 July	29,031	7198	5098	1.19 (1.15–1.23)
	Sri Lanka ODI Durham^d^	1 day; 29 June	7025	4935	3047	
	Sri Lanka ODI Oval^d^	1 day; 1 July	11,363	8637	5744	
	Sri Lanka ODI Bristol^d^	1 day; 4 July	6273	5553	4013	
	Pakistan ODI Lords^d^	1 day; 10 July	20,732	2067	1366	
	Pakistan ODI Edgbaston^d^	1 day; 13 July	17,624	6493	1417	
	Grosvenor Park^c^	4 days; 14–17 July	291	57	57	
	Silverstone F1^d^	5 days; 14–18 July	105,931	18,803	13,022	
	Pakistan T20 Trent Bridge^d^	1 day; 16 July	14,208	8990	5626	
	RFL Challenge Cup^d^	1 day; 17 July	25,441	7963	5289	
	Pakistan T20 Headingley^d^	1 day; 18 July	16,850	442	297	
Outdoor partially structured	Open Golf^d^	8 days; 11–18 July	32,000	61,148	39,123	1.07 (1.03–1.12)
Outdoor unstructured	Goodwood^d^	4 days; 8–11 July	33,954	7747	4600	1.95 (1.90–2.00)
	Latitude^e^	4 days; 22–25 July	37,437	28,846	19,933	
	Tramlines^e^	3 days; 23–25 July	39,041 unique individuals	16,182	12,884	

*Abbreviations*: *ODI* One Day International cricket match, *RFL* Rugby Football League, *T20* Short format cricket match, *UKHSA* United Kingdom Health Security Agency

^a^Obtained from https://docs.google.com/spreadsheets/d/10qsaRZMgeMYKENUU2Crs6OfdSotRhs54Kgj_QiVfHO8/edit?usp=sharing

^b^musical/opera theatre

^c^theatre comedy

^d^sporting event

^e^popular music/arts festival

The main outcome of interest was a positive COVID-19 test, as recorded in the NHS Test and Trace Second Generation Surveillance System. At the time these events occurred, national guidance on testing recommended that individuals who had new onset of the cardinal COIVD-19 symptoms test by polymerase chain reaction (PCR) as soon after onset as possible. Whilst awaiting a result, they were required to self-isolate. The general public over the age of 12 years were advised to undertake twice weekly lateral flow device (LFD) tests, though the uptake was low [4]. Additionally, there were other national testing programmes, e.g. PCR testing of individuals prior to elective care, and more intensive asymptomatic testing regimes of care home staff. Results from private providers were not included in SGSS data. Positive results, whether obtained via a lateral flow device (LFD) or a PCR test, were used for the primary analysis. Any recorded negative result via either LFD or PCR was considered a control outcome.

Covariates of interest were attendee age (in 5-year bands), sex, self-reported COVID-19 vaccine status, and weekly estimated regional prevalence of COVID-19 using figures local to the attendee, and irrespective of age, available from the Office for National Statistics.

### Study design

The nature of such a large-scale events programme meant conventional study designs, such as a cohort approach, would be challenging, both in collecting comprehensive data on all participants, and in identifying an appropriate control group. We therefore used a self-controlled case series (SCCS) design, in which each participant acts as their own control [5]. Inclusion in a SCCS is conditional on having the outcome of interest during a predefined observation period. The observation period is divided into high and baseline risk periods, and an incident rate ratio is calculated comparing the rate of the outcome during these two periods. The method relies on two key assumptions. Firstly, the occurrence of the outcome should not affect the likelihood of subsequent exposure. In this instance, a positive COVID-19 test would preclude attendance at an event, and so we applied an extension of SCCS methodology where observation begins at the point of exposure [6]. This extension is valid for exposures that are unique within the observation time of interest, which is reasonable during the 18-day period after each event (i.e. that unique event could not be attended again). The second assumption is that the outcome of interest should not substantially curtail observation. This assumption tends to be invalid for outcomes that are frequently fatal. COVID-19 in this context is only fatal in rare instances, though in any case, the extension we applied also negates this assumption.

**Fig. 1:**
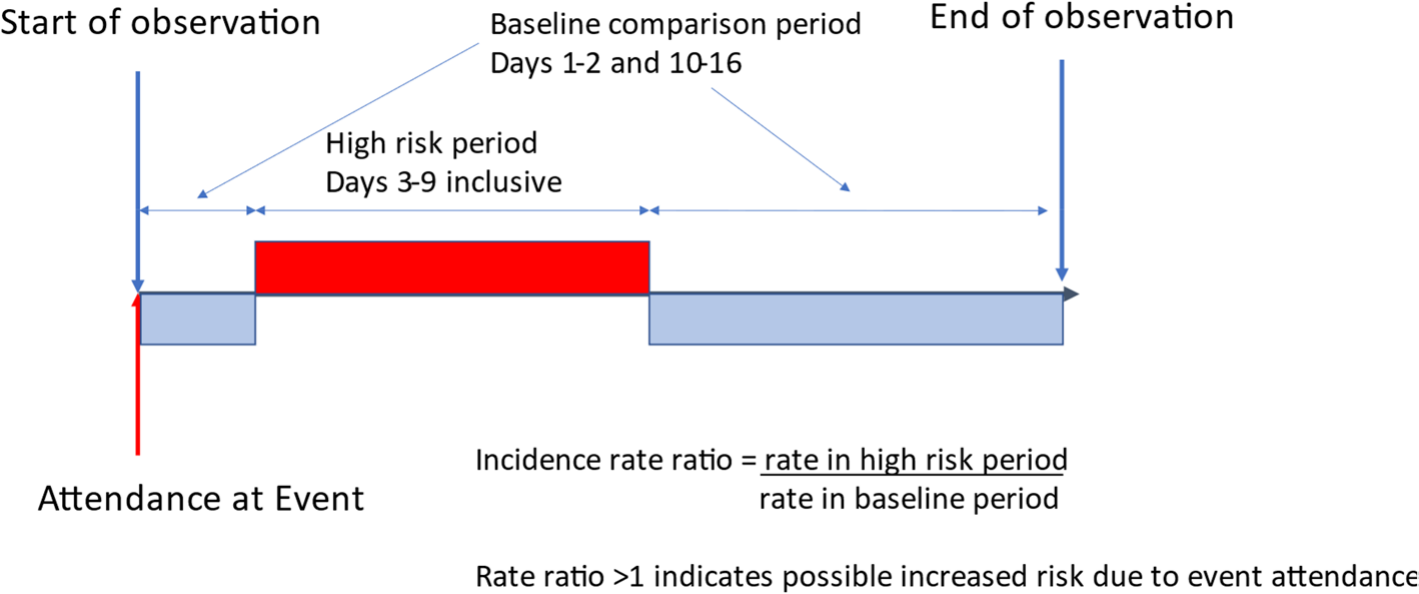
Individual participant study timeline

The key advantage of an SCCS design is that each participant acts as their own control, which means that characteristics that do not vary over the observation period cannot confound any associations. It is also a statistically efficient study design, not requiring a high absolute risk of the outcome to be able to detect an increased risk. Risk factors for the outcome that do vary over the observation period can be adjusted for in the analysis. Figure 1 outlines the key features of the SCCS design we used.

### Statistical analysis

Using conditional Poisson regression, a within-person incidence rate ratio was calculated comparing the rate of positive testing for SARS-CoV-2 during high risk and baseline periods of time. The high risk period was days 3 to 9 following event attendance, and the baseline period was days 1 and 2 and 10–16 following the event when infection detection was assumed to be unaffected by attendance. For events where data indicated multi-day attendance (Goodwood, Tramlines, Latitude, Silverstone, Open Golf), the first day of the high risk period was defined as either 3 days after the first attendance date or as the final attendance date if this was > 3 days after the first attendance date. Rate ratios were adjusted for estimates of underlying regional SARS-CoV-2 prevalence to account for population level fluctuations in infection risk. Prevalence was categorised as < 200, 200–< 300, 300–< 400, 400–< 500, 500–< 600, > 600 per 10,000, with data shown in Additional File 1 Table S1. Each day of the observation period was then categorised according to the prevalence in the region of the attendee on that day, and prevalence was included in the regression model as a categorical variable. A rate ratio of 1 suggests that SARS-CoV-2 infection risk was unaffected by attendance at the event, whilst a rate ratio and 95% confidence interval all greater than 1 suggests transmission risk may have been increased by attendance.

We anticipated that the probability of attendees testing themselves for SARS-CoV-2 would be highest immediately after the event and would likely fall over the observation period. This would mean that positive cases were more likely to be detected in the high risk period than in the baseline. To account for this, we also calculated the rate ratio for negative test results, using the same risk and baseline periods as in the primary analysis. We used this as a proxy for testing behaviour and subsequently calculated a ratio of incidence rate ratios (IRR) (IRR for positive tests/IRR for negative tests), to account for any declining testing probability over the study period.

### Sensitivity analyses

The following sensitivity analyses were conducted: (1) the timing of high risk periods was extended to days 3–11 after the event, with days 1 and 2 and 12–18 acting as the baseline; (2) the definition of a positive test was restricted to first PCR positive test; and (3) at the request of referees, we stratified by individual outdoor unstructured event (Goodwood, Latitude and Tramlines).

## Results

Events for which data were available are shown in Table 1, along with the estimated total number of attendees where known. For multi-day events, the total number of unique attendees across all days was not known, so instead, the total number of event days and the maximum number of attendees on a single day is given.

In total, data were available for 188,851 phase 3 ERP event attendees, amongst whom 3357 people registered a positive test for COVID-19 in NHS Test and Trace in the 18 days after the event. Table 2 shows the breakdown of this population by age, sex, and broad category of event attended. Outdoor unstructured events had a younger age profile (74% aged < 30 years) and a higher proportion of female attendees (47%) than other event types. By contrast, nearly 50% of outdoor seated event attendees were aged > 40 years, and 85% were male. Most COVID-19 cases were in attendees of the outdoor unstructured events *n* = 2012, with a much smaller number in attendees of indoor seated events (*n* = 30). Self-reported vaccination status varied by event type ranging from 11% of outdoor unstructured event attendees reporting one or more doses to 65% reporting one or more doses among outdoor seated event attendees.

**Table 2:** Characteristics of event attendees with positive tests in 18 days post-event

	**All events**	**Indoor seated**	**Outdoor seated**	**Outdoor partially structured**	**Outdoor unstructured**
People testing positive by LFT or PCR (*N*)	3357	30	783	532	2012
Positive by PCR only (*N*)	2716	21	621	459	1615
People testing negative (*N*)	37,707	970	14,531	9863	12,305
Age band *N* (%)					
10–14	102 (3)	0	10 (1)	*	88 (4)
15–19	888 (26)	0	46 (6)	23 (4)	819 (41)
20–24	470 (14)	*	77 (10)	33 (6)	356 (18)
25–29	439 (13)	10 (33)	121 (15)	88 (17)	220 (11)
30–34	243 (7)	7 (23)	87 (11)	83 (16)	66 (3)
35–39	151 (5)	0	59 (8)	53 (10)	39 (2)
40–44	225 (7)	0	99 (13)	48 (9)	78 (4)
45–49	198 (6)	*	63 (8)	41 (8)	91 (5)
50–54	243 (7)	*	82 (10)	55 (10)	104 (5)
55–59	161 (5)	*	49 (6)	47 (9)	63 (3)
60–64	89 (3)	0	37 (5)	31 (6)	21 (1)
65–69	46 (1)	0	27 (3)	17 (3)	*
70–74	16 (0)	*	10 (1)	*	*
75–79	7 (0)	0	*	*	0
80–84	*	0	*	*	0
Missing	76 (2)	0	12 (2)	0	64 (3)
Male	2040 (61)	20 (67)	619 (79)	471 (89)	1064 (53)
Female	1309 (39)	10 (33)	164 (21)	61 (11)	940 (47)
Missing	8 (0)	0	0	0	8 (0)
Self-reported vaccine status					
Unvaccinated	2294 (68)	15 (50)	95 (15)	314 (59)	1457 (72)
> = 1 dose	396 (12)	5 (17)	508 (65)	63 (12)	213 (11)
Missing	667 (20)	10 (33)	160 (20)	155 (29)	342 (17)

^*^Cells with < 5 counts redacted to maintain participant anonymity

Crude IRRs with 95% confidence interval (CI) for testing positive for SARS-CoV-2 during days 3–9 following event attendance compared with days 1 and 2 and 10–16 aggregated at event-type level were as follows: 1.29 (0.63–2.63) for indoor seated, 3.31 (2.97–3.68) for outdoor unstructured, 1.35 (1.17–1.55) for outdoor seated, and 1.07 (0.90–1.27) for outdoor partially structured.

**Fig. 2:**
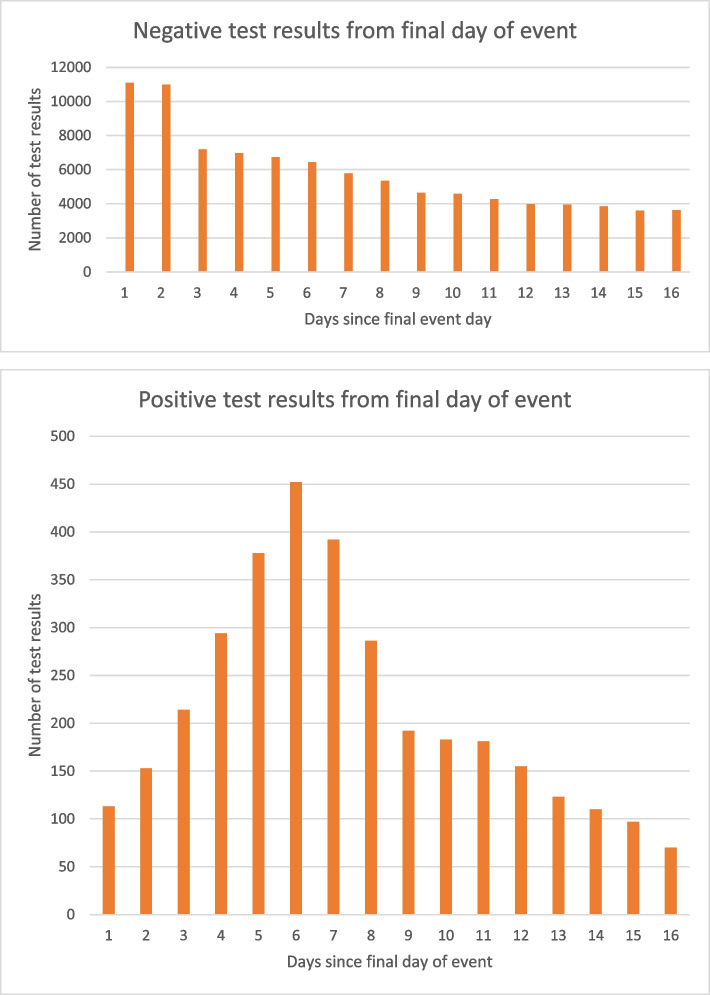
Trends in negative and positive COVID-19 test results over the observation period

Trends in recording of negative tests were calculated for each event type, with incident rate ratios and 95% confidence intervals for the same comparison periods as follows: 1.00 (0.90–1.10) for indoor seated, 1.95 (1.90–2.00) for outdoor unstructured, 1.19 (1.15–1.23) for outdoor seated, and 1.07 (1.03–1.12) for outdoor partially structured. Figure 2 shows the decline in reporting of negative tests over the observation period, alongside the trend for positive tests over the same period.

After accounting for the trends in negative testing behaviour over the same observation period, and SARS-CoV-2 prevalence, incident rate ratios were reduced. Rate ratios with 95% confidence intervals were as follows: 1.16 (0.53–2.57) for indoor seated, 1.70 (1.52–1.89) for outdoor unstructured, 1.12 (0.95–1.30) for outdoor seated, and 0.65 (0.51–0.83) for outdoor partially structured (Table 3).

**Table 3:** Association between event attendance and testing positive for COVID-19

Event type People testing positive *N*	Positive test during baseline	Positive test during high risk period	Crude rate ratio	Adjusted rate ratio^a^ (95% CI)
**Indoor seated (Piccadilly Theatre, Leeds Grand, The Grange)**				
30	15	15	1.29 (0.63–2.63)	1.16 (0.53–2.57)
**Outdoor unstructured (Tramlines, Latitude, Goodwood)**				
2012	469	1543	3.31 (2.97–3.68)	1.70 (1.52–1.89)
**Outdoor seated (Cricket, RFL Challenge Cup, Grosvenor Park, Silverstone, Wimbledon)**				
783	380	403	1.35 (1.17–1.55)	1.12 (0.95–1.30)
**Outdoor partially structured (Open Golf)**				
532	285	247	1.07 (0.90–1.27)	0.65 (0.51–0.83)

*Abbreviations*: *RFL* Rugby Football League

^a^Adjusted for regional prevalence and accounting for trends in testing over the observation period by dividing the rate ratio for positive tests by the rate ratio for negative tests over the same period

Results of all sensitivity analyses were broadly in line with the main findings (see Additional File 1, Tables S1, S2 and S3).

## Discussion

Using a self-controlled design, we evaluated whether attendance at a wide range of sport and cultural events in England during the summer of 2021 was associated with an increased risk of infection with SARS-CoV-2. We found no evidence for an increased risk associated with attendance at indoor seated, outdoor seated, and outdoor partially seated events. By contrast, attendance at outdoor, multi-day, unstructured events (Goodwood, Latitude and Tramlines) was associated with a 70% increased risk of transmission. For context, the risk of infection in the baseline period was ~0.9% for Latitude attendees in the study; a 70% increase would take this risk to 1.53%.

The strengths of this work include the large number and varied nature of events included, which is, to our knowledge, the most comprehensive studied to date. Our use of a self-controlled design means that potential confounding factors that may differ between individuals (e.g. occupation or general approach to risk taking) are implicitly dealt with in the design.

However, the study was not a randomised trial, meaning that care needs to be taken with any causal interpretation. Whilst time fixed confounders could not affect the results, time varying factors that are associated with infection risk could have impacted the findings. Variations in background SARS-CoV-2 prevalence is one factor we were able to control for, but behavioural changes, e.g. reduced socialising after the event due to financial constraints, could not be accounted for. Similarly, any substantial geographical variation in predominant SARS-CoV-2 variant could be a time varying confounder if variant type was strongly linked with transmissibility.

Data on attendance and infection status were not available for all event attendees. The mechanisms for data unavailability are (1) the event organiser did not have or make available the attendee data; (2) the attendee did not test at all in the observation period; (3) the attendee tested but did not report their result. Given the self-controlled nature of our study design, it would be necessary for those with missing data to have a systematically different relative risk of becoming infected at the event, compared with those for whom we had data. As this is not testable or verifiable, we acknowledge that any inferences we make are based on the assumption that unavailability of testing data is not strongly related to the individual’s personal relative risk of infection at the event.

The precise timing and location of infection acquisition was not known. Therefore, any increased risk associated with event attendance includes infections acquired both at the event itself but also due to event-related activities (e.g. travel to and from the event or socialising before/after). Our analysis was unable to distinguish the impact of each of these separate aspects of event attendance.

Testing bias over the observation period is likely, with an increased probability of testing and reporting test results in the period shortly after an event, coinciding with our pre-specified high risk period. This would tend to overestimate any increased risk of infection associated with the event but our adjustment for this using the pattern of negative testing results should mitigate this bias. Notably, we observed different patterns of negative testing according to the type of event, with rate ratios for negative testing varying from 1.00 for indoor seated events to 1.95 for outdoor unstructured events. This meant that in final adjusted models, changes from the crude estimate were driven mainly by regional prevalence for some event types (indoor seated and outdoor partially structured) and more by negative testing trends for others (outdoor unstructured and outdoor seated).

The precision of our estimates is low in some settings, particularly for indoor seated events. Whilst the point estimate of 1.16 does not clearly suggest these events were associated with an increased risk, the wide confidence intervals with an upper bound of 2.57 mean we also cannot rule out a potentially large increased risk, including a risk commensurate with that seen in the outdoor unstructured events. However, a separate study of air quality carried out in theatres taking part in the ERP found that under the conditions of the ERP events, air quality was sufficiently high to suggest transmission risk would be low [8].

The generalisability of our findings may be limited as they provide evidence of infection risk under the specific conditions at the time the ERP was operating. This includes the background of pre-event mitigation strategies, such as testing and advice to avoid events if symptomatic. Other event-related factors including individual/crowd behaviour at events, ventilation of event space, time spent at an event, likelihood of attendees being infected, mode of travel to and from the event, age distribution of attendees, and vaccination coverage may also have affected infection risk. Separately, the prevailing epidemiological circumstances will also affect generalisability, e.g. dominance of the Delta variant, background prevalence, vaccination rates, and societal restrictions/behaviours. However, the impact of varying some of these factors has been modelled by others and may not always lead to large differences in the relative risk of infection [7].

We were unable to study how vaccination impacts on infection risk in these settings because our inclusion criteria and analysis were conditional on having a positive test result. In order to study the impact of vaccination, we would need to use a different study design and have data available on all attendees and their vaccination status, in order to see if the vaccinated were less likely to become infected.

We categorised events with some degree of similarity in their nature but acknowledge there remains heterogeneity within categories. For example, there may have been important differences in venue, crowd size, and individual attendee behaviour between Grosvenor Park Outdoor Theatre and the Rugby League Challenge Cup at Wembley Stadium. Nonetheless, we did not detect any material differences between the results for individual events within each category and chose to present the combined results to improve precision. Similarly, we acknowledge that the description of each category does not capture their entire nature. For example, outdoor seated events generally have indoor hospitality and bathroom facilities, meaning attendees may also spend large amounts of time indoors at these events, particularly if the event itself is held over a long period.

### Findings in context

Other UK ERP studies investigated both air quality and behavioural risk factors at events [8, 9]. Air quality was measured through carbon dioxide levels, with high levels indicating poor ventilation. The majority of venues had good air quality throughout, but peaks were noted in areas of high congestion, allowing organisers to focus on areas where ventilation improvements were needed. Behavioural studies found wide variation in the ability of attendees to physically distance at an event and in face covering usage. These were largely driven by venue size, event capacity, and whether mitigations such as wearing a face covering were mandatory rather than discretionary.

Over the last 12 months, others have evaluated transmission risks associated with a variety of events, using a range of methodologies. Their findings largely coincide with our results.

Indoor live concerts were studied through randomised trials in both Barcelona and Paris, with all those undergoing randomisation required to test negative shortly before the event. Neither found any increased risk of infection associated with attendance [10, 11]. However, pre-event testing and mask wearing were mandatory, and the total number of cases detected was very small. By contrast, a non-randomised cohort analysis found that attendees of festivals in Catalonia had a twofold increased risk of infection compared with non-attendees [12]. Shukla et al. [13] also found a strong corelation between attendance at the Kumbh Mela and local infection rates. A non-randomised study in March 2021 amongst 4584 indoor full-capacity concert attendees in Barcelona found that after pre-concert screening, only six attendees were diagnosed with COVID-19 within 2 weeks of attending the concert [14].

Smith et al. studied the UK ERP using data from contact tracing to see how ERP event attendance and subsequent infection risk varied [15]. Some events were found to have higher rates of positive case attendance and onwards transmission; Euros attendees were more likely to be infected on entry and to be associated with onwards transmission than attendees at other events such as Wimbledon. Sociocultural and mitigating factors were highlighted as being potentially very different between events.

These findings are largely reassuring as there was little evidence of an increased risk of SARS-CoV-2 transmission after attendance at most event types, in the context of a carefully managed event programme where pre-attendance negative testing was required and where venues had taken some mitigating steps to reduce the likelihood of transmission. Attendance at the three festivals we studied was associated with a 70% increased risk of transmission. The majority of infected festival attendees were under 50 years and unvaccinated at the time of the event. It is unlikely that the additional cases acquired at these events would have led to substantial morbidity for those directly infected, and they would be unlikely to have a large impact on wider community incidence compared with the number of cases occurring outside the festival setting. This study also highlights a novel use of the self-controlled case series design which could be used in future for monitoring the impact of single event attendance on infection risk, requiring only data on cases who attended the event, along with information about general testing trends amongst all attendees. Whilst the events we studied were all cultural, the method would be equally effective at a broader range of events, e.g. international conferences or summits. What is essential is the collection of sufficient data to permit the relevant analysis, namely attendance at the event and subsequent testing results amongst all attendees, regardless of result. We recommend this be factored into privacy notices for event attendance in the context of any future important epidemic situation where evidence of risk from event attendance is needed.

## Conclusions

We found no evidence of an increased risk of SARS-CoV-2 associated with attendance at the majority of events and venue types included in the 2021 phase 3 UK ERP and for which we had data. By contrast, attendance at outdoor unstructured multi-day events was associated with a 70% increased risk of transmission. These results may not generalise to all SARS-CoV-2 epidemiological contexts and were obtained from events where attendance was based on self-declared SARS-CoV-2 negative status at entry. They also highlight the utility of a self-controlled design to study infection risk associated with event attendance.

### Supplementary Information

**Additional file 1. **Results for all sensitivity analyses (Table S1, S2, S3) and the detailed regional COVID-19 prevalence data used in the analysis (Table S4). **Table S1.** Regional SARS-CoV-2 prevalence figures (per 10,000) over study period only. **Table S2.** Association between event attendance and testing positive for COVID-19, restricted to PCR testing. **Table S3.** Association between event attendance and testing positive for COVID-19, extending risk period to days 3-11 and baseline period to days 1,2 and 12-18. **Table S4.** Association between event attendance and testing positive for COVID-19, for individual outdoor unstructured events.

## Data Availability

The datasets used and/or analysed during the current study are available from the corresponding author on reasonable request.

## Abbreviations

CI: Confidence interval
DCMS: Department for Culture, Media and Sport
ERP: Events Research Programme
IRR: Incidence rate ratio
LFD: Lateral flow device
LFT: Lateral flow test
NHS: National Health Service
PCR: Polymerase chain reaction
SCCS: Self-controlled case series
UKHSA: United Kingdom Health Security Agency
