# Supplementary material for: A self-controlled case series study to measure the risk of SARS-CoV-2 infection associated with attendance at sporting and cultural events: the UK Events Research Programme events
Source: BMC Med. 2024 Mar 6;22:100. doi: 10.1186/s12916-024-03276-4 (PMC10918946; doi:10.1186/s12916-024-03276-4)
Supplement: Supplementary file 1 — Additional file 1. Results for all sensitivity analyses (Table S1, S2, S3) and the detailed regional COVID-19 prevalence data used in the analysis (Table S4). Table S1. Regional SARS-CoV-2 prevalence figures (per 10,000) over study period only. Table S2. Association between event attendance and testing positive for COVID-19, restricted to PCR testing. Table S3. Association between event attendance and testing positive for COVID-19, extending risk period to days 3-11 and baseline period to days 1,2 and 12-18. Table S4. Association between event attendance and testing positive for COVID-19, for individual outdoor unstructured events. [file 12916_2024_3276_MOESM1_ESM.docx]

**Content:**

**Table S1:** Regional SARS-CoV-2 prevalence figures (per 10,000) over study period

**Table S2:** Association between event attendance and testing positive for COVID-19, restricted to PCR testing only

**Table S3:** Association between event attendance and testing positive for COVID-19, extending risk period to days 3-11 and baseline period to days 1,2 and 12-18

**Table S4:** Association between event attendance and testing positive for COVID-19, for individual outdoor unstructured events

**Table S1 Regional SARS-CoV-2 prevalence figures (per 10,000) over study period**

| **Date** | **England** | **East Midlands** | **East of England** | **London** | **North East** | **North West** | **South East** | **South West** | **West Midlands** | **Yorkshire and The Humber** |
| --- | --- | --- | --- | --- | --- | --- | --- | --- | --- | --- |
| 28/06/2021 | 190.8 | 141.5 | 99.8 | 154.8 | 393.3 | 350.4 | 125.6 | 149.4 | 177.3 | 239.5 |
| 29/06/2021 | 207.5 | 157.1 | 110.7 | 167.8 | 437.9 | 368.4 | 139.8 | 161 | 194.1 | 262.7 |
| 30/06/2021 | 223.5 | 171.2 | 121.6 | 179.8 | 481.8 | 385.8 | 152.9 | 172.3 | 212.4 | 282.8 |
| 01/07/2021 | 239.2 | 188.1 | 134.2 | 192.1 | 526.2 | 400.5 | 165.5 | 183 | 229.2 | 302.5 |
| 02/07/2021 | 252 | 200.9 | 145.9 | 201.7 | 555.4 | 412.8 | 175.9 | 192.2 | 245.9 | 317.3 |
| 03/07/2021 | 264.4 | 212.6 | 158 | 212.2 | 589.8 | 421.3 | 186.1 | 201.7 | 259.9 | 333 |
| 04/07/2021 | 275.5 | 226.3 | 169.4 | 220.1 | 620.3 | 428.8 | 192.8 | 211.5 | 272.4 | 348.2 |
| 05/07/2021 | 287.2 | 241.1 | 183.4 | 230.6 | 662.4 | 427 | 202.7 | 218.7 | 284.2 | 366.5 |
| 06/07/2021 | 301.8 | 257.2 | 198.7 | 241.8 | 704.8 | 433.5 | 212.9 | 231.2 | 300.7 | 389 |
| 07/07/2021 | 319.7 | 276.1 | 215.4 | 257.4 | 748.9 | 445.4 | 225.2 | 246.3 | 316.8 | 419.1 |
| 08/07/2021 | 329.7 | 288.3 | 226.3 | 263.7 | 773.7 | 448.7 | 232.5 | 258.9 | 328.2 | 433.3 |
| 09/07/2021 | 341.6 | 301.5 | 237.6 | 271.5 | 804.1 | 455.7 | 242 | 272.6 | 339.3 | 450.7 |
| 10/07/2021 | 349.9 | 312.2 | 243.3 | 277.7 | 825.3 | 460.7 | 248.2 | 280.9 | 348.9 | 462.4 |
| 11/07/2021 | 359.8 | 322 | 252.7 | 285 | 842.5 | 466.1 | 258.1 | 295 | 361.8 | 472.3 |
| 12/07/2021 | 377.6 | 344.1 | 268.7 | 297.4 | 864 | 478.6 | 273.4 | 319.4 | 386.4 | 492.1 |
| 13/07/2021 | 397.7 | 365.8 | 290.3 | 311.3 | 886 | 494 | 291.8 | 348.1 | 410.6 | 512.2 |
| 14/07/2021 | 425.4 | 396.4 | 324.2 | 333.3 | 906.9 | 514.8 | 321.7 | 385.4 | 442.4 | 534.9 |
| 15/07/2021 | 473.2 | 440.9 | 373.9 | 378.2 | 956.2 | 553.9 | 369.7 | 443 | 495.5 | 581.9 |
| 16/07/2021 | 511.5 | 479.9 | 413 | 416.2 | 985.3 | 585.5 | 407.1 | 488.8 | 538.2 | 620.6 |
| 17/07/2021 | 528.7 | 498 | 435.7 | 436.1 | 978 | 598.5 | 425.6 | 512.2 | 556.1 | 634.5 |
| 18/07/2021 | 538.4 | 509.5 | 449.7 | 449.4 | 960.9 | 602.6 | 438.1 | 527.1 | 565.3 | 643.1 |
| 19/07/2021 | 543.4 | 515.1 | 460.6 | 461.9 | 939.6 | 603.9 | 446.4 | 533.7 | 563.7 | 646.2 |
| 20/07/2021 | 530.6 | 502.8 | 455.9 | 459.9 | 882.1 | 585.4 | 442.4 | 521.9 | 544.9 | 627.1 |
| 21/07/2021 | 498.3 | 474.5 | 429.3 | 440.9 | 802.3 | 544.6 | 417.3 | 489.5 | 510.2 | 588.3 |
| 22/07/2021 | 446.6 | 429.4 | 385.8 | 403 | 702.6 | 489.1 | 371.3 | 432.4 | 451.5 | 532.2 |
| 23/07/2021 | 400.4 | 384.8 | 346.9 | 368 | 618.1 | 437.6 | 331.5 | 387.5 | 400.2 | 478.1 |
| 24/07/2021 | 374.2 | 360.3 | 324.3 | 346.5 | 570.5 | 409.7 | 309.4 | 361.1 | 369.3 | 450.1 |
| 25/07/2021 | 350.7 | 339.3 | 303.1 | 328.8 | 526.6 | 385.9 | 288.5 | 338.8 | 343.1 | 420.9 |
| 26/07/2021 | 321.5 | 312.4 | 278 | 306.2 | 462.4 | 350.8 | 263.6 | 317.3 | 311.2 | 385.9 |
| 27/07/2021 | 304.2 | 303.8 | 262.2 | 292.9 | 422.9 | 326.4 | 248.5 | 306.2 | 291.1 | 367.6 |
| 28/07/2021 | 295.9 | 303.6 | 255.2 | 283.9 | 397.4 | 314.4 | 242.1 | 305.7 | 278.2 | 359.2 |
| 29/07/2021 | 291.7 | 305.7 | 250.2 | 279.2 | 374.1 | 306.6 | 240.3 | 309.6 | 269.2 | 358.8 |
| 30/07/2021 | 287.5 | 308.2 | 248.9 | 272.7 | 357 | 298.5 | 238.9 | 306.7 | 260.9 | 358.8 |
| 31/07/2021 | 284.2 | 311.4 | 248.1 | 267.7 | 339.5 | 290.3 | 236.2 | 307.6 | 258.1 | 358.1 |
| 01/08/2021 | 282.4 | 312.7 | 246.9 | 265.5 | 325.7 | 284.9 | 235.8 | 307.8 | 255.7 | 359.9 |
| 02/08/2021 | 282.5 | 316.8 | 248.6 | 264.6 | 307 | 283.6 | 238.4 | 308.1 | 257.4 | 361.5 |
| 03/08/2021 | 284.3 | 321.3 | 251.3 | 266 | 296.5 | 283.9 | 240.9 | 313.5 | 260.2 | 362.6 |
| 04/08/2021 | 287.7 | 325.5 | 253.9 | 269.5 | 292.7 | 285 | 245.1 | 316.8 | 265.3 | 368.7 |
| 05/08/2021 | 292.5 | 332.9 | 258.8 | 273.7 | 293.1 | 288.6 | 249.5 | 321.7 | 274.9 | 371 |
| 06/08/2021 | 297.3 | 339.4 | 261.3 | 277.7 | 289.7 | 292.3 | 253.9 | 331.5 | 281.8 | 376.3 |
| 07/08/2021 | 299.9 | 340.6 | 261.8 | 281.4 | 290.3 | 294.6 | 258.1 | 335.8 | 284.3 | 378.3 |
| 08/08/2021 | 302.5 | 344.7 | 264 | 283.8 | 290.4 | 296.6 | 261.4 | 340.1 | 287.9 | 379 |
| 09/08/2021 | 305.4 | 349.4 | 266.4 | 282.8 | 291.9 | 298.2 | 264.4 | 347.3 | 295.1 | 380.9 |
| 10/08/2021 | 306.4 | 348.5 | 264.7 | 281.3 | 288.7 | 301.6 | 268.1 | 347.5 | 299 | 382.1 |
| 11/08/2021 | 306.8 | 346.1 | 264.6 | 281.9 | 283 | 303.5 | 273.1 | 348.1 | 302.5 | 375.2 |
| 12/08/2021 | 305.8 | 346.2 | 264.7 | 278.9 | 277 | 301.1 | 275.6 | 348.5 | 302.4 | 371.5 |

**Table S2: Association between event attendance and testing positive for COVID-19, restricted to PCR testing only**

| Event Type  People testing positive N | Positive test during baseline | Positive test during high risk period | Crude Rate Ratio | Adjusted Rate Ratio* (95%CI) |
| --- | --- | --- | --- | --- |
| **INDOOR SEATED (Piccadilly Theatre, Leeds Grand, The Grange)** | | | |  |
| 21 | 12 | 9 | 0.96 (0.41-2.29) | 0.80 (0.30-2.14) |
| **OUTDOOR UNSTRUCTURED (Tramlines, Latitude, Goodwood)** | | | |  |
| 1,615 | 376 | 1,239 | 3.30 (2.94-3.71) | 1.69 (1.50-1.92) |
| **OUTDOOR SEATED (Cricket, RFL Challenge Cup, Grosvenor Park, Silverstone, Wimbledon)** | | | |  |
| 621 | 301 | 320 | 1.35 (1.16-1.58) | 1.14 (0.96-1.36) |
| **OUTDOOR PARTIALLY STRUCTURED (Open Golf)** | | | |  |
| 459 | 247 | 212 | 1.06 (0.88-1.28) | 0.66 (0.55-0.80) |

*adjusted for regional prevalence and accounting for trends in testing over the observation period by dividing the rate ratio for positive tests by the rate ratio for negative tests over the same period.

**Table S3: Association between event attendance and testing positive for COVID-19, extending risk period to days 3-11 and baseline period to days 1,2 and 12-18**

| Event Type  People testing positive N | Positive test during baseline | Positive test during high risk period | Crude Rate Ratio | Adjusted Rate Ratio* (95%CI) |
| --- | --- | --- | --- | --- |
| **INDOOR SEATED (Piccadilly Theatre, Leeds Grand, The Grange)** | | | |  |
| 33 | 12 | 21 | 1.75 (0.86-3.56) | 1.60 (0.73-3.50) |
| **OUTDOOR UNSTRUCTURED (Tramlines, Latitude, Goodwood)** | | | |  |
| 2,095 | 341 | 1,754 | 4.01 (3.57-4.51) | 1.97(1.73-2.22) |
| **OUTDOOR SEATED (Cricket, RFL Challenge Cup, Grosvenor Park, Silverstone, Wimbledon)** | | | |  |
| 802 | 351 | 501 | 1.41 (1.23-1.62) | 1.20 (1.03-1.39) |
| **OUTDOOR PARTIALLY STRUCTURED (Open Golf)** | | | |  |
| 575 | 279 | 296 | 1.02 (0.87-1.20) | 0.50 (0.40-0.64) |

*adjusted for regional prevalence and accounting for trends in testing over the observation period by dividing the rate ratio for positive tests by the rate ratio for negative tests over the same period.

**Table S4: Association between event attendance and testing positive for COVID-19, for individual outdoor unstructured events**

| Event Type  People testing positive N | Positive test during baseline | Positive test during high risk period | Crude Rate Ratio | Adjusted Rate Ratio* (95%CI) |
| --- | --- | --- | --- | --- |
| **TRAMLINES** | | | |  |
| 915 | 197 | 718 | 3.65 (3.11-4.27) | 1.89 (1.59-2.25) |
| **LATITUDE** | | | |  |
| 1,049 | 255 | 794 | 3.11 (2.70-3.59) | 1.44 (1.24-1.67) |
| **GOODWOOD** | | | |  |
| 48 | 17 | 31 | 2.04 (1.13-3.68) | 1.55 (0.74-3.21) |
